# White Kidney Bean (*Phaseolus Vulgaris* L.) Consumption Reduces Fat Accumulation in a Polygenic Mouse Model of Obesity

**DOI:** 10.3390/nu11112780

**Published:** 2019-11-15

**Authors:** Elizabeth S. Neil, John N. McGinley, Vanessa K. Fitzgerald, Corey A. Lauck, Jeremy A. Tabke, Madyson R. Streeter-McDonald, Linxing Yao, Corey D. Broeckling, Tiffany L. Weir, Michelle T. Foster, Henry J. Thompson

**Affiliations:** 1 Cancer Prevention Laboratory, Colorado State University, Fort Collins, CO 80523, USA; elizabeth.neil@colostate.edu (E.S.N.); john.mcginley@colostate.edu (J.N.M.); vanessa.fitzgerald@colostate.edu (V.K.F.); corey.lauck@rams.colostate.edu (C.A.L.); jeremy.tabke@rams.colostate.edu (J.A.T.); mady.streeter-mcdonald@colostate.edu (M.R.S.-M.); 2 Proteomics and Metabolomics Facility, Colorado State University, Fort Collins, CO 80523, USA; linxing.yao@colostate.edu (L.Y.); corey.broeckling@colostate.edu (C.D.B.); 3 Department of Food Science and Human Nutrition, Colorado State University, Fort Collins, CO 80523, USA; tiffany.weir@colostate.edu (T.L.W.); michelle.foster@colostate.edu (M.T.F.)

**Keywords:** *Phaseolus vulgaris*, white kidney bean, adiposity, obesity, gut health, *Akkermansia muciniphila*, Firmicutes to Bacteroidetes ratio, bile acids, farnesoid X receptor

## Abstract

Clinical studies indicate that eating common bean, *Phaseolus vulgaris* L., plays a role in body weight regulation but mechanisms have yet to be elucidated. Here, we investigated the anti-obesogenic activity of white kidney bean in a mouse model of dietary-induced obesity. Bean consumption reduced the accumulation of adipose tissue in male and female C57BL6 mice. The anti-obesogenic effect of white kidney bean was not due to alterations in energy intake, energy excreted in the feces, or feed efficiency ratio. While bean consumption increased the mass of the intestine, no marked differences were consistently observed in crypt height, mucin content of goblet cells, proliferation index or zone of proliferation. However, significantly higher concentrations of total bacteria and of *Akkermansia muciniphila* were detected in cecal content of bean-fed mice, and the ratio of Firmicutes to Bacteroidetes was reduced. Bile acid content was higher in the ileum of bean-fed mice, but transcript levels of farnesoid X receptor were not significantly affected. Whether changes in bile-acid-mediated cell signaling play a role in bean-related differences in fat accumulation and/or overall metabolic health requires further investigation.

## 1. Introduction

The global pandemic of obesity is associated with increased incidence of and mortality due to cardiovascular disease, type-2 diabetes, and certain types of cancer [1,2]. While pharmacological and surgical approaches to control obesity have been pursued aggressively, the global impact of such interventions is limited by constraints on access and by cost [3]. Investigations of patterns of food consumption indicate a possible alternative may be diet because certain populations manage to maintain desired body weight despite living in obesogenic environments [4]. Recent meta-analyses indicate that consumption of grain legumes, i.e., common bean, chickpea, dry pea, and lentil, also referred to as pulses [5], is associated with improved weight management relative to populations in which the consumption of pulses is low [6,7]. Given the complexity of obesity, we recently sought to determine whether the impact of pulses on body weight observed in prospective clinical studies could be reproduced under the controlled conditions that are possible to achieve with the use of preclinical rodent models [8]. Therefore, we utilized common bean, the predominant pulse consumed in clinical studies, in our initial rodent investigations in rats [6,7]. In those models, white kidney bean, a specific type of common bean, markedly reduced visceral adiposity in female rats resistant and susceptible to dietary-induced obesity [8]. 

The work reported herein was designed to extend the work done in rats by determining whether white kidney bean consumption would reduce accumulation of lipid in adipocytes in female and/or male mice in a widely used polygenic model of obesity in C57BL6 mice (B6). This study had three primary objectives: (1) determine if white kidney bean inhibited the development of obesity irrespective of the sex of the animal; (2) assess whether the effect of white kidney bean on adiposity was independent of differences in caloric intake; and (3) examine bean specific effects on (a) energy excretion in the feces, (b) concentration of specific microbiota in the cecum, and (c) the content of bile acid metabolites and activity of a bile acid receptor signaling transcription factor, farnesoid X receptor (FXR) since this transcription factor and its downstream targets have been reported to influence a number of metabolic pathways involved in energy balance [9,10,11]. 

## 2. Materials and Methods 

### 2.1. Experimental Animals 

For Experiment 1, 90 C57BL6/J mice, 49 male and 41 female mice weaned from our in-house breeding colony were used. Our in-house breeding colony founders were obtained from Jackson Laboratories (Bar Harbor, ME, USA). For Experiment 2, 16 eleven-week-old male NCI C57BL6/NCr mice were obtained from Charles River Laboratories NCI (Frederick, MD, USA). These inbred strains of C57BL6 mice were fed a purified high fat diet (60% dietary Kcal as fat) obesogenic diet formulation (D12492 high fat diet; Research Diets, Inc., New Brunswick, NJ [12,13]). Mice were maintained on a 12 h light/dark cycle at 27.5 ± 2 °C with 30% relative humidity and given *ad libitum* access to diet and distilled water until they were randomized to experimental groups. All animal studies were performed in accordance with the Colorado State University Institutional Animal Care and Use Committee, protocol 18-7746.

### 2.2. Experimental Diets

The formulation of the experimental diets and the rationale for bean concentration have been published [14,15]. Diet formulations are detailed in Table 1. Briefly, the diets were formulated to match macronutrient levels (i.e., protein, carbohydrate and crude fiber) across the experimental groups. The saturated fat source used in the experimental diets was palm oil since the fatty acid composition is more consistent than that of lard. The level of white kidney bean was (40% w/w), which is about twice as high as median intakes of pulse consuming subgroups in the US and Canada. However, the dietary level is actually similar to dietary amounts consumed in developing countries where pulses are dietary staples [15]. White kidney bean was grown in field plots maintained at Colorado State University, Fort Collins, CO. The bean seed was cooked and freeze-dried as previously reported [8]. The freeze-dried bean was then milled into a homogeneous powder and stored at −20 °C until incorporated into diets. Diets were formulated using specific guidelines [16], and adjusted using the proximate analysis (IEH-Warren Laboratory, Greeley, CO, USA) of the cooked whole bean powder as previously reported [8].

### 2.3. Experimental Design

#### 2.3.1. Experiment 1

**Figure 1:**
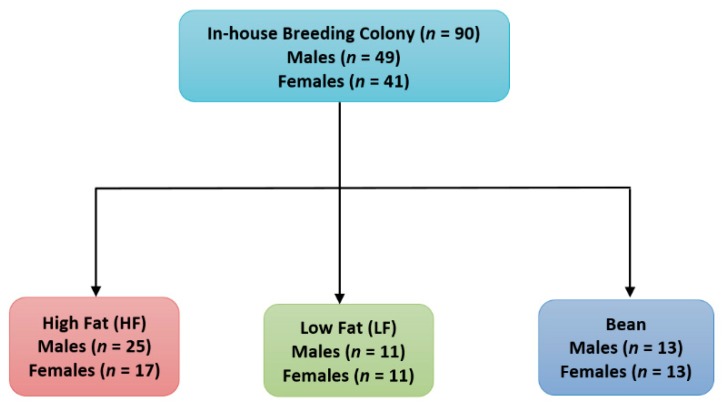
Experiment 1 flow chart.

The first experiment was designed to address the question of whether bean consumption affected weight regulation irrespective of gender, age, duration of bean consumption, or degree of susceptibility for obesity, a situation with parallels to cross-sectional population studies. For this experiment, 90 C57BL6/J mice, 49 male and 41 female mice from our in-house breeding colony were serially assigned at weaning, by sex, to one of three diet groups. Mice were maintained on experimental diets for a period of 13.4 (range 12.6 to 14.7), 19.8 (range 19.6–19.9) or 31.5 (range 27.1 to 33.6) weeks. Mice were fed either a high-fat (HF; males *n* = 25, females *n* = 17) control diet, low-fat (LF; males *n* = 11, females *n* = 11) control diet or high-fat diet to which freeze-dried cooked, white kidney bean powder had been added (Bean HF; males *n* = 13, females *n* = 13), as shown in Figure 1. It should be recognized that the high-fat diet is the positive control that promotes the development of obesity and the low-fat group is the negative control, i.e., mice with obesogenic potential fed a low-fat diet that slows the rate at which obesity develops. Mice were group housed and fed their respective diets ad libitum throughout the experiment. Body weight data was collected weekly. The experiment was terminated serially between 12 to 34 weeks of feeding experimental diets.

The study endpoints were body weight adjusted to tibia length which is a proxy for body mass index in human populations and weight gain. Subcutaneous and visceral fat mass were also evaluated. For subcutaneous fat, the inguinal fat pad was assessed since changes in that adipose depot have been reported to account for obesity susceptibility in B6 mice [17]. For visceral fat, the mesenteric depot was evaluated since it is associated with metabolic syndrome. Fat mass was also normalized to tibia length, as recommended in reference [18].

#### 2.3.2. Experiment 2 

In this energy balance experiment that used a paired feeding technique, we assessed whether the effect of white kidney bean on adiposity was independent of differences in caloric intake and bean specific effects on energy excretion in the feces. Sixteen eleven-week-old male NCI C57BL6/NCr mice obtained from Charles River Laboratories NCI (Frederick, MD, USA) were randomized to one of two diet groups and fed either HF control diet (*n* = 8) or Bean HF diet (*n* = 8). These were the same diets as those used in Experiment 1. Mice were individually housed in wire mesh bottomed cages for feces collection. Each cage was equipped with a polycarbonate resting platform on the cage floor and modified tunnel feeders in order to accurately measure food intake. These devices were designed and fabricated in our laboratory (Supplementary Figure S1). Fresh pre-measured food was given daily and mice were allowed to acclimate to the caging system and feeding approach for 21 days, after which, the study commenced. To initiate the experiment, mice were paired by body weight and the 8 pairs were randomized to either the high-fat control or bean high-fat diet. On a daily basis, each bean-fed mouse was given *ad libitum* access to diet. Within a pair, the amount of food consumed by the bean-fed mouse was given to its paired control diet fed partner over the subsequent 24 h period. Paired feeding was continued for the 84 day duration of the study. Body weight and food consumption data were collected daily. Feces were collected daily for the last 7 days of the experiment. Fecal material was analyzed for total concentration of energy using a bomb calorimeter (Model 1261 Isoperibol Bomb Calorimeter, Parr Instrument Company, Moline, IL, USA).

### 2.4. Necropsy

For both Experiment 1 and 2, all the animals in each group were subjected to the following procedures. Mice were not fasted prior to necropsy. Mice were anesthetized using isoflurane inhalation and subsequently euthanasied by cervical dislocation. The gastrointestinal tract including the stomach to the superior portion of the rectum was removed. The small intestine was cut at the ileocecal junction and weighed with the stomach attached. A 6.5 cm length of ileum superior to the cecum was excised and ileum contents were collected in a 2.0 mL cryovial by flushing the lumen using a sterile gavage needle and syringe with 1.5 mL of sterile saline and the tube snap-frozen in liquid nitrogen for metabolomic analysis. Gavage needles were sterilized in a hot bead sterilizer between animals. A 1 cm section of flushed ileum immediately superior to the cecum was cut and placed in 10% neutral buffered formalin. The remaining ileum was laid on a Kimwipe and a midline incision was made down the entire long axis using blunt tipped scissors. The ileum was reflected, and the edges of the serosa gently tacked down to the Kimwipe. While holding one end of the ileum securely with Adson forceps, a polished stainless-steel spatula was used to gently scrape the ileum mucosa in one continuous motion; contents were placed into a cryovial and snap-frozen in liquid nitrogen for subsequent RNA analysis. The cecum was cut from the colon, weighed and snap-frozen in liquid nitrogen. The colon was rinsed in sterile saline and fecal pellets extruded while maintaining proper anatomic orientation. Sections of colon (1 cm) representing the ascending, transverse and descending portions was excised and fixed in 10% neutral buffered formalin. Inguinal subcutaneous and abdominal visceral adipose tissue were harvested and weighed. In addition, a small portion of these adipose depots were excised and fixed in 10% neutral buffered formalin. Remaining portions of fat depots were snap-frozen in liquid nitrogen. All snap-frozen tissues were stored at −70 °C. The left tibia of each animal was removed, cleaned and measured in mm using a pair of digital calipers in order to normalize the tissue weights. All formalin fixed tissues were processed for histological evaluation.

### 2.5. RNA Transcript Expression

RNA was isolated from frozen ileum using the RNeasy Mini kit (Qiagen, Germantown, MD, USA) according to the manufacturer’s protocol. Isolated RNA purity (260/280 and 260/230 ratios) and concentration was checked via NanoDrop (Thermo Fisher Scientific, Waltham, MA, USA). RNA integrity was determined using a Bio-Rad Experion (Bio-Rad, Hercules, CA, USA). cDNA was synthesized from 1 µL of total RNA using Superscript II reverse transcriptase (Invitrogen, Carlsbad, CA, USA). DNA oligo primers were synthesized (Integrated DNA Technologies, Coralville, IA, USA) [19]. The primer sequences used are shown in Table 2. Quantitative real-time PCR was performed on an iCycler (Bio-Rad, Hercules, CA, USA) with optical-grade 96-well plates (Thermo Fisher Scientific, Waltham, MA, USA). Each 20 µL reaction mixture was composed of 5 µL of water, 10 µL 2X SYBR green Supermix (Bio-Rad, Hercules, CA, USA), 1.5 µL of forward and reverse primers at a final concentration of 0.3 µM, and 2 µL of synthesized cDNA. The following PCR conditions were used: 95 °C for 3 min, followed by 40 cycles of 95 °C for 15 s and 56.5 °C for 1 min. Fluorescent products were detected at the last step of each cycle. qPCR is followed by a melting curve analysis to verify primer specificity under the following conditions: 80 cycles starting at 57 °C for 10 s and increasing by 0.5 °C each cycle. Each sample was run in triplicate and the relative expression of FXR, small heterodimer partner (SHP) and fibroblast growth factor 15 (FGF15) were calculated by normalizing the threshold cycle (Ct) values to 18S. The fold change in expression relative to the control-fed animals was computed by the delta, delta Ct method as described [20].

### 2.6. Bacterial Quantification by qPCR

An established approach was used to assess levels of specific bacterial populations. DNA oligo primers coding for 16S rRNA sequences unique to *Akkermansia muciniphila*, and bacteria in the phyla Firmicutes and Bacteroidetes, or common to all bacteria were used for qPCR analysis. The DNA oligomers were synthesized by Integrated DNA Technologies, Coralville, IA, and are shown in Table 2 [21,22,23,24].

Frozen cecums were removed from the freezer three at a time and allowed to slightly thaw on the bench top, approximately 5–10 min. Small scissors were used to make an incision in the cecum and the mucosa was reflected. A clean, polished stainless-steel spatula was used to remove approximately 100 mg of cecal contents and placed in a dry bead tube and DNA extracted according to the manufacturer’s protocol, QIAamp PowerFecal DNA kit (Qiagen, Germantown, MD, USA). The isolated DNA was checked for purity (260/280 and 260/230 ratios) and concentration via NanoDrop (Thermo Fisher Scientific, Waltham, MA, USA).

qPCR was performed on cecal DNA using a modified version of the protocol described [23]. Briefly, qPCR amplification and detection were performed using an iCycler (Bio-Rad, Hercules, CA, USA) with optical-grade 96-well plates (Thermo Fisher Scientific, Waltham, MA, USA). Each 20 µL reaction mixture was composed of 5 µL of water, 10 µL 2X SYBR green Supermix (Bio-Rad, Hercules, CA, USA), 1.5 µL of forward and reverse primers at a final concentration of 0.3 µM, and 2 µL of template DNA (10 ng/µL). PCR conditions were as follows: 95 °C for 3 min, followed by 40 cycles of 95 °C for 15 s, 60 °C for 30 s, and 72 °C for 30 s, and a final extension at 72 °C for 5 min. Fluorescent products were detected at the last step of each cycle. qPCR is followed by a melting curve analysis to verify primer specificity under the following conditions: 80 cycles starting at 55 °C for 10 s and increasing by 0.5 °C each cycle. Each sample was run in triplicate, Ct values were normalized to 16S (926 Forward (Fwd.), 1062 Reverse (Rev.)), and the fold change in expression relative to the control was computed by the delta, delta Ct method as described [20].

### 2.7. Histology

#### 2.7.1. Histology and Image Acquisition

The tissue fixed in 10% neutral buffered formalin for 24 hours was then processed through a series of graded ethanols, cleared in toluene, embedded in paraffin and sections cut at 4 µM. Sections were stained with H&E, alcian blue nuclear fast red (AB) [25], and Ki-67 immunostaining as described previously [26]. Images of stained intestine were captured at 200× and images of adipose tissue at 100× magnification using a Zeiss AxioCamHR (Zeiss, Thornwood, NY, USA) digital camera mounted on a Zeiss Axioskop II microscope. Prior to capturing images, background shading correction was achieved using the Zeiss AxioVision software. Images were captured (1300 × 1030 pixels, 24 bit RGB, 150 DPI) and saved as JPEG files.

#### 2.7.2. Morphometric Analysis

Adipocyte area measurement was performed as previously described [8]. A total of ten crypts in ileum, and ascending, transverse and descending colon were analyzed from each animal across serial sections of three different stains listed in Section 2.7.1. Crypts were chosen from multiple image fields along the length of each segment in order to decrease bias and selection was based on the following criteria: (1) a single layer of epithelial cells visible from the base of the crypt to the top of the of villus; (2) patent crypt lumen; (3) visible crypt lamina propria. Ileum and colon crypt height was measured manually from the base of the crypt to the brush border of the uppermost enterocyte nearest the lumen in H&E stained images using the caliper function in Image Pro Plus v4.5 (Media Cybernetics, Inc. Rockville, MD, USA). AB stained images depicting crypt goblet cells and intra-crypt mucin were processed using the built-in alcian blue vector in the colour deconvolution plugin for ImageJ v1.52d (National Institutes of Health (NIH), Bethesda, MD, USA), resulting in three separate color channel images, red, green and blue. A macro was written in Image Pro Plus v4.5 (Media Cybernetics, Inc. Rockville, MD, USA) to analyze the AB images. Briefly, the blue channel image was imported along with the original AB image. The blue channel image was converted to 8-bit grayscale (256 shades of gray) and contrast applied, resulting in a black and white image. Crypts were circumscribed one at a time on the original AB image as an area of interest (AOI). The AOI was copied from the original AB image, pasted to the corresponding XY coordinates in the black and white image and the segmentation range was set 0–254, i.e., black representing alcian blue stained mucin within the crypt against a white (255) background. The software measured the total sum black area in µM^2^ of the AOI and exported the value automatically via dynamic data exchange to an Excel spreadsheet for analysis. To assess cell proliferation, Ki-67 staining was performed as previously described [26] and analyzed using the immunoratio plugin for ImageJ [27]. 

### 2.8. Bile Acid Analyses 

#### 2.8.1. Sample Preparation

Ileum lavage samples were lyophilized and 20 mg of dried samples were suspended in 1.2 mL of 80% methanol in water, mixed for 2 h at 4 °C, followed by centrifugation at 17,000 g × 15 min at 4 °C. The supernatants were recovered and the solvent was removed under nitrogen until the samples were completely dry. The dried extracts were resuspended in 0.15 mL 80% methanol [28]. 

#### 2.8.2. LC–MS

A quantity of 3 μL of extract was injected into a Waters Acquity UPLC system and separated using a Waters Acquity UPLC HSS T3 (1.8 µM, 1.0 × 100 mm), using a gradient from solvent A (water, 0.1% formic acid) to solvent B (acetonitrile, 0.1% formic acid). Injections were made in 100% A, held at 100% A for 1 min, ramped to 98% B over 12 min, held at 95% B for 3 min, and then returned to starting conditions over 0.05 min and allowed to re-equilibrate for 3.95 min, with a 200 µL/min constant flow rate. The column and samples were held at 40 °C and 6 °C, respectively. The column eluent was infused into a Waters Xevo G2 Q-TOF-MS with an electrospray source in negative mode, scanning 50–1200 m/z at 0.2 seconds per scan, alternating between MS (6 V collision energy) and MSE mode (15–30 V ramp). Calibration was performed using sodium iodide with 1 ppm mass accuracy. The capillary voltage was held at 2200 V, source temp at 150 °C, and nitrogen desolvation temp at 350 °C with a flow rate of 800 L/hr.

#### 2.8.3. Metabolite Data Processing

Our procedure has been reported [29,30]. Briefly, for each sample, raw data files were converted to .cdf format, and matrix of molecular features as defined by retention time and mass (m/z) was generated using XCMS software in R for feature detection and alignment. The centWave algorithm used for feature detection of LC–MS data. Features were grouped using RAMClustR, with normalization set to ‘none’. LC–MS data were first annotated by searching against an in-house spectra and retention time database using RAMSearch. RAMClustR was used to call the findMain function from the interpretMSSpectrum package to infer the molecular weight of each LC–MS compound and annotate the mass signals. The complete MS spectrum and a truncated MSE spectrum were written to a .mat format for import to MSFinder. The MSE spectrum was truncated to only include masses with values less than the inferred M plus its isotopes, and the .mat file precursor ion is set to the M + H ion for the findMain inferred M value. These .mat spectra were analyzed to determine the most probable molecular formula and structure. MSFinder was also used to perform a spectral search against the MassBank database. All results were imported into R and a collective annotation is derived with prioritization of MSFinder mssearch >MSFinder structure >MSFinder formula >findMain M. All R work was performed using R version 3.3.1 [6].

### 2.9. Statistical Analyses 

Experiment 1. Data were normally distributed. Body mass and fat mass data were subjected to factorial analysis of variance (ANOVA) with diet group and sex as the factors and time on diet and age as covariates. Experiment 2. Body mass, morphometric, bacterial, and RNA transcript expression data were evaluated by one way ANOVA or the Kruskal–Wallis test, depending on data distribution (D’Agostino and Pearson omnibus normality test). For both experiments, the Fisher or Dunn’s post hoc test, respectively, was used depending on data distribution. Differences were considered significant with p ≤ 0.05. Data analyses were conducted using Systat, version 13.0 (Systat Software, Inc., San Jose, CA, USA), SAS, version 9.2 (SAS Instituite, Cary, NC, USA), and Graph Pad Prism, version 5.2 (GraphPad Software, Inc., La Jolla, CA, USA).

## 3. Results

### 3.1. Effect of ad Libitum Feeding of White Kidney Bean in Male and Female B6 Mice 

#### 3.1.1. Effect of Bean Consumption in an Unselected Population of B6 Mice

**Figure 2:**
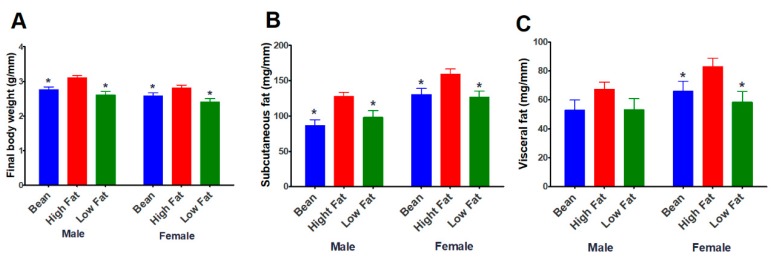
Effect of bean consumption on body weight and adiposity (Experiment 1). (**A**) final body weight; (**B**) subcutaneous fat; (**C**) visceral (mesenteric) fat; Units are g or mg of mass divided by length of tibia in mm. Factorial ANOVA with time on diet (TOD) as a covariate. Final body weight: sex *p* = 0.0012, diet *p* < 0.0001, interaction *p* = 0.6952, TOD, not significant (ns); Subcutaneous fat: sex *p* < 0.0001, diet *p* < 0.0001, interaction *p* = 0.6186, TOD, ns; Visceral (mesenteric) fat: sex *p* = 0.0417, diet *p* = 0.0058, interaction *p* = 0.7208, TOD, ns; Post hoc comparisons, * different relative to high-fat diet within gender (*p* ≤ 0.05).

White kidney bean incorporated into the high-fat diet, reduced obesity susceptibility. Body mass indexed to tibia length, accumulation of lipid in subcutaneous and visceral adipose depots in bean-fed mice was similar to values observed in mice fed the low-fat diet (Figure 2).

Female mice have larger subcutaneous and visceral fat pads than males when data was normalized to tibia length. It appeared that the effect of bean was greater on the subcutaneous fat pad of both male and female mice. 

### 3.2. Effect of Bean Energy Balance Using a Paired Feeding Experimental Design

In Experiment 2, pair feeding was utilized to determine if bean consumption exerts a specific effect on the feed efficiency ratio, energy excreted in the feces or fat mass. To address these questions, an 84 day paired feeding study was completed in B6 male mice consuming a high-fat diet (60% kcal from fat) plus or minus white kidney bean. Metabolic cages adapted for mice were used to permit quantitative assessment of food intake and of excreted feces.

#### 3.2.1. Energy Balance

The mice in the control group were slightly underfed (not statistically significant) because of the strict paired-feeding methodology; there was no compensation for the small amount of food spilled, about 150 mg/day. Despite that difference, the feed efficiency ratio did not vary by diet group. Daily fecal collections were subjected to bomb calorimetry. There were no differences between groups in either concentration of energy in the feces or total energy excreted in the feces (Table 3).

#### 3.2.2. Body Composition 

Final body weight and body mass indexed to tibia length were the same across groups, which was the intended outcome of the paired feeding study design (Table 4). A difference in the assessment of visceral fat in this study versus the study reported in Section 3.1.1 was that three visceral fat pads were assessed to ascertain whether bean exerted differential effects on these fat depots. Quantitatively, the greatest reduction was in the subcutaneous fat depot (28% reduction) with a 12% reduction in visceral adipose depot mass.

**Figure 3:**
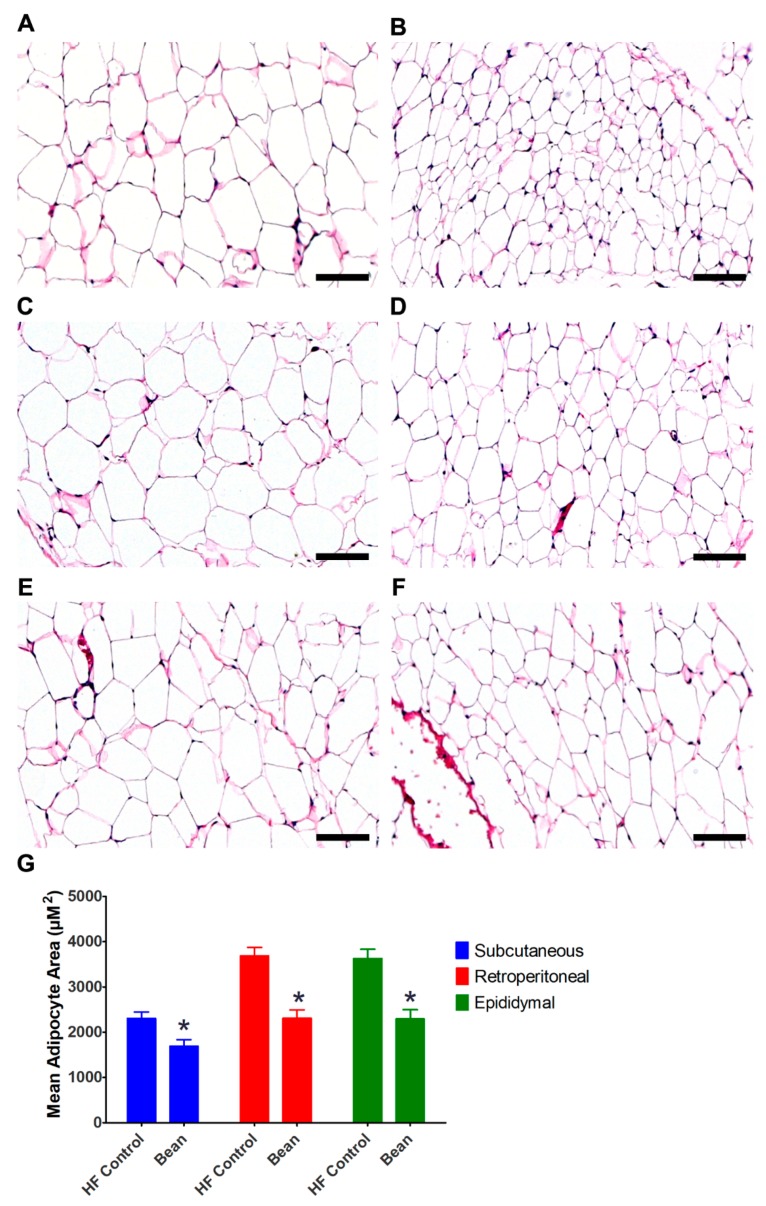
Representative H&E stained sections of fat depots (Experiment 2). (**A**) HF Control subcutaneous fat; (**B**) Bean subcutaneous fat; (**C**) HF Control retroperitoneal fat; (**D**) Bean retroperitoneal fat; (**E**) HF Control epididymal fat; (**F**) Bean epididymal fat; (**G**) Adipocyte morphometry; ANOVA: subcutaneous adipocyte area *p* = 0.0094, retroperitoneal adipocyte area *p* = 0.0001, epididymal adipocyte area *p* = 0.0004; * different relative to control diet (*p* ≤ 0.05); all images 100 × magnification, bars = 100 µM; HF Control *n* = 8, Bean *n* = 8; HF: high fat.

Recognizing the value of morphometric analysis of adipocyte size as a complementary approach to measuring fat mass, the inguinal, retroperitoneal and epididymal fat pads were evaluated (Figure 3). The results not only affirmed the mass data, but strengthen the observation that bean consumption decreased lipid accumulation in both subcutaneous and visceral fat depots.

#### 3.2.3. Evaluation of the Intestinal Tract 

**Figure 4:**
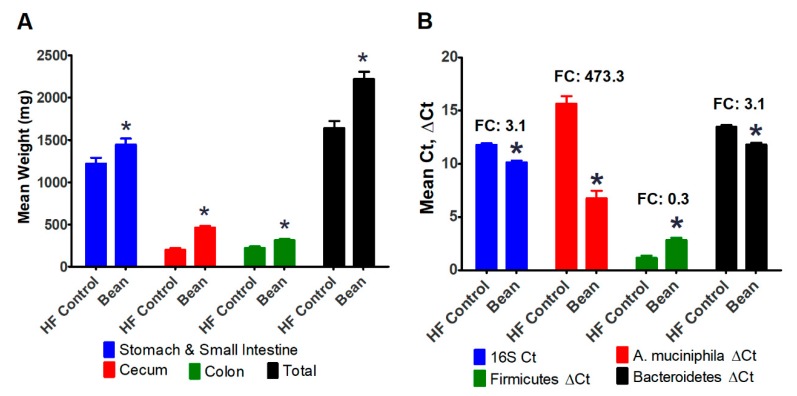
Effects of paired-feeding of white kidney bean on intestinal mass and cecal bacterial populations (Experiment 2). (**A**) Intestinal weights; (**B**) Cecal bacteria populations; ANOVA: Stomach Small Intestine p = 0.0343, Cecum p < 0.0001, Colon p = 0.0027, Total p = 0.0003; 16S Ct *p* = 0.0001, *A. muciniphila* ∆Ct *p* < 0.0001, Firmicutes ∆Ct *p* = 0.0004, Bacteroidetes ∆Ct *p* = 0.0001; * different relative to HF control diet (*p* ≤ 0.05); Control *n* = 8, Bean *n* = 8; FC: fold change; HF: high fat.

During the necropsy process in the experiment reported in Section 3.1, there was visual evidence that the intestinal tract of bean-fed mice was larger than control mice despite the fact that body size was smaller. Therefore, in the paired feeding experiment, each segment of the intestinal tract was removed and weighed (Figure 4A). There was an overall increase in the size of the intestine with the most pronounced effect on the cecum and colon.

Because the cecum was markedly enlarged, and this phenotype is also observed in germ-free mice, we decided to determine whether the concentration of microbes in the cecum was affected by bean consumption. Using primers for 16S rRNA that are common to all bacteria, marked enhancement of bacterial content was observed in the cecum of bean-fed mice (Figure 4B). Based on other reports, we further probed the DNA isolated from the cecal content and found a 473-fold increase in the content of *A. muciniphila*, a bacterium known to digest mucin and that is associated with health benefits. We also measured the cecal concentration of bacteria in the phyla Firmicutes and Bacteroidetes and found the ratio to be reduced in bean-fed mice.

### 3.3. Structural and Functional Assessments

#### 3.3.1. Morphometric Analyses 

Given that the size of the intestinal tract was enlarged, experiments were performed to determine whether the changes in size were accompanied by changes in morphology. For this purpose, our analyses were focused on ileum, and the ascending, transverse and descending segments of the colon. Morphometric analyses were conducted to determine crypt height, the amount of goblet cell alcian-blue-positive staining, and the Ki-67 proliferative index (Table 5). Overall, there were some differences in crypt height, but little evidence of consistent difference in either alcian blue staining or the Ki-67 proliferative index. Further analysis of the zone of proliferation also failed to detect significant differences associated with bean treatment (data not shown).

#### 3.3.2. Bile Acid and FXR Signaling 

Because of the observed changes in the Firmicutes to Bacteroidetes ratio, we reasoned that FXR signaling and bile acid metabolism might be affected in the ileum [10,11,19,31]. Because we had expended our repository of tissue from Experiment 2, specimens from Experiment 1 were evaluated. Ileal lavage of a randomly chosen subset of animals from Experiment 1 (HF control *n* = 3, Bean *n* = 3) was subjected to metabolomic analysis to provide an overview of changes induced. A total of 35 ions were annotated as bile acids and total signal associated with white kidney bean consumption was increased 2.4-fold relative to control. Four ions were identified to be different between treatment groups: 3,7-Dihydroxy-12-oxocholanoic acid (*p* = 0.02, 2.2-fold change), sulfolithocholylglycine (*p* = 0.057, 2.2-fold change), taurocholic acid (*p* = 0.09, 4.56-fold change), and N-[(3a,5b,7a)-3-hydroxy-24-oxo-7-(sulfooxy)cholan-24-yl]-glycine (*p* = 0.077, 2. 9-fold change). 

Since receptors for these bile acids exist in the ileum [10,11], the transcript levels of one of the receptors that has also been linked to energy balance was measured. FXR transcript levels and expression of FXR downstream targets (SHP and FGF15) were not different between groups (data not shown).

## 4. Discussion

Preclinical models of human diseases afford the opportunity to deconstruct complex clinical observations, but it is important to consider the extent to which the findings observed in one model are likely to reflect what occurs in human populations, especially with a polygenic disease like obesity [32]. To address this concern, it is now recommended that more than one preclinical model be used to examine a specific relationship and it is considered advantageous to use multiple animal species when that is feasible. Thus, the work reported herein was designed to determine whether white kidney bean consumption would reduce body fat mass in a widely used polygenic model of obesity in C57BL6 mice (B6) as it did in the Levin rat model of polygenic obesity [8]. Male and female mice were protected against susceptibility to obesity and both visceral and subcutaneous fat depots were reduced in size (Experiment 1, Figure 2). While only female rats were investigated in the study reported in [8], the findings presented herein extend those results showing that bean has anti-obesogenic effects in male as well as female mice and at a dietary dose of bean that was 47% lower than fed in the rat studies. These findings are consistent with the recently reported meta-analysis of 22 prospective studies of pulse consumption [6,7] and support the value of mechanistic experiments in preclinical models. Our findings also indicate the relevance of public health strategies designed to increase consumption of pulses such as common bean, which are under utilized staple food crops [33], in mounting an effort to combat the pandemic of obesity. 

Because body weight was reduced in bean-fed mice in Experiment 1, a paired feeding study was conducted in male B6 mice. The paired feeding approach permitted us to determine whether anti-obesogenic activity was due to reduced food intake, perhaps related to increased satiety that has been proposed as a mechanism of improved weight regulation in clinical trials. This experiment also afforded the opportunity to examine the effects of bean consumption on energy balance since speculation from prospective clinical trials suggests that improved body weight regulation in response to bean consumption is due to either decreased food efficiency ratio and/or increase fecal energy excretion [6,7]. It appears that the reduction in fat pad mass observed in both Experiments 1 and 2 can not be explained by these commonly proposed mechanisms: improved satiety: anti-obesogenic activity was observed when food intake was actually higher in bean-fed mice (Table 3); decreased energy efficiency: the food efficiency ratio was unaffected; or increased fecal energy excretion: neither mass of feces or fecal energy concentration were affected. Therefore, other hypotheses were explored.

A number of experiments in the B6 diet induced obesity (DIO) model have reported that the development of obesity is associated with changes in the ecology of the gut microflora and that obesity can be reversed by fecal transplantation techniques, a finding consistent with the microbiome playing a causal role [34]. It has also been reported that consumption of various common bean containing diets causes a shift in the ecology of the gut microbiome [35]. When those two observations were “overlaid”, it prompted our specific hypotheses that 1) at the phylum level, the bean would alter the ratio of Firmicutes to Bacteroidetes, and 2) that there would be an increase in the content of *A. muciniphila*. The relevance of shifts in the Firmicutes to Bacteroidetes ratio is that bacteria in these phyla affect the type of bile salts in the intestinal lumen via their bile salt hydrolase activity [9,10,11]. Depending on the nature of the bile acids secreted into the intestine and microbial metabolism of those compounds, the mixture of bile acid metabolites with agonistic and antagonistic effects on bile acid receptors such as FXR can be affected. The relevance of *A. muciniphila*, is that colonization of the gut microbiome with this bacterium has been reported to be inversely associated with obesity, diabetes, and inflammation [36,37,38,39,40,41].

The data in Figure 4 are consistent with a bean-specific effect on these components of the gut microbiome. We note that the relationship between obesity and *A. muciniphila* is an area of active investigation and that the relationship between obesity and ratio of Firmicutes to Bacteroidetes is controversial [24,42]. The fact that the differences in microbial content and adiposity were correlated supports: (1) studies designed to test causality, and (2) a broader understanding of changes in microbiome ecology and how it impacts host function. Because of the changes in bacterial populations that were observed, we also investigated intestinal morphometry. Other than the increased crypt height found in the transverse and descending segments of the colon of bean-fed mice, no consistent differences were observed, indicating the lack of structural or functional differences as reflected by these commonly assessed endpoints. However, it remains to be determined how other measures of gut function are affected. These findings are consistent with recently reported work and point to the need for studies related to inflammation, endotoxemia, and immune function [35,43,44,45,46].

We have previously reported that hepatic transcript expression of cholesterol 7α-hydroxylase (Cyp7α1) is induced by feeding common bean [47], suggesting that bile acid metabolism is affected. The changes in Cyp7α1 also imply that FXR signaling is involved since Cyp7α1 is a downstream target of FXR regulation. As a first step in tackling the complexity of bile acid metabolism, the luminal content of the ileal segment of the intestine was profiled for bile acid metabolites. We focused on this intestinal segment since it is recognized to play a central role in bile-acid-mediated signaling and it is proximal to the cecum where white kidney bean feeding was found to induce changes in the microbiome that have been reported to affect bile acid metabolism [10,11,19,31,48]. Overall, the signal intensity associated with bile-acid-related ions was approximately 2-fold higher in bean-fed mice. Of the four ions identified in this study, two are products of microbial metabolism (sulfolithocholylglycine (*p* = 0.057, fold change = 2.2) and N-[(3a,5b,7a)-3-hydroxy-24-oxo-7-(sulfooxy)cholan-24-yl]-glycine (*p* = 0.077, 2.9 fold change) and one has been reported to antagonize FXR signaling (taurocholic acid (*p* = 0.09, 4.56 fold change). However, functionally, we observed FXR transcript levels to be modestly higher, but the difference was not statistically significant. Moreover, transcript levels of two of FXR’s downstream targets were unaffected. Given that various bile acids are agonists or antagonists of FXR, it appears that white kidney bean’s effect is not selective. Additional work is required to determine whether white kidney bean exerts system effects via the modulation of bile acid metabolism. 

Of the additional observations made in this study that will merit future investigation, it is important to highlight the fact that white kidney bean had a greater effect on the subcutaneous versus visceral fat depots. This observation not only suggests that limiting fat quantification to the visceral fat pads diminishes the likelihood of detecting overall bean-mediated effects on fat accumulation, but more importantly, the fact that the subcutaneous fat depot is impacted by white kidney bean consumption is consistent with energy dissipation via the uncoupling of oxidative phosphorylation in adipocytes, at least in B6 mice. With regard to this hypothesis, several points merit comment. Adipocytes in subcutaneous depots have developed evolutionarily to play a critical role in thermoregulation associated with protection of the organism against cold stress. Accordingly, white adipocytes in subcutaneous depot have a great deal of plasticity in terms of energy metabolism and undergo bidirectional transcription to beige adipocytes [49]. In addition, as noted above, B6 mice are obesity-resistant for the first 8 weeks of life, at which point a developmental switch appears to be activated that decreases the ratio of beige to white adipocytes in the subcutaneous fat compartment such that the animal becomes obesity-susceptible [17]. This potential to become obese is promoted by positive energy balance which is accelerated when a high-fat diet is fed beginning at 8 weeks of age [50], an effect that bean consumption appears to have reversed. Thus, the regulation of the developmental pathway affecting obesity susceptibility in B6 mice provides a focus for future mechanistic studies.

There are translational limitations that need to be considered. The goal of the work reported herein was to deconstruct the clinical observation that pulse consumption is inversely associated with obesity in order to propel public health efforts to promote the consumption of common bean and other pulses as a feasible, cost-effective approach for the reduction of obesity and the chronic disease with which obesity is associated. However, there are many questions that must be addressed. One obvious question is whether all types of common bean are created equal relative to anti-obesogenic activity and/or their effects on the gut and its associated flora. Of equal importance is whether all pulses share the same health attributes as common bean. Moreover, it is critical to establish the dose above which consumption of beans like white kidney bean must be consistently maintained to achieve health benefits. Similarly, while common bean and other pulses are somewhat unique in that the whole cooked seed can be used as a food ingredient, there is value in identifying the chemical components of the bean that have bioactivity so that they are not inadvertently lost during food processing. Of course, identifying bioactives requires knowledge of their targets. Therefore, more mechanistic studies are required and arguably, this is most readily done using pre-clinical models. 

## 5. Conclusions

Meta-analyses of 22 prospective clinical studies of pulse consumption indicate that pulses play a role in body weight regulation. Consistent with our previous report in two rat models of obesity resistance and obesity susceptibility, we show that white kidney bean consumption reduces accumulation of fat in both visceral and subcutaneous adipose depots [8]. This study extends the previous work to another species, mice using a strain (C57BL6) that is recognized to be sensitive to DIO and to male as well as female animals. It also shows that the effect of bean is observed when bean provides 40% of dietary protein. In our previous work, bean provided 75% of dietary protein. In addition, using a paired feeding approach, we confirm not only is the anti-obesogenic effect unrelated to alterations in feed efficiency ratio, but it is independent of effects on energy intake or energy excreted in the feces. While bean consumption increased the mass of the intestine, no marked differences were observed in the morphology of the intestine in terms of crypt height, mucin content, Ki-67 proliferation index or zone of proliferation. However, we did detect markedly higher content of bacteria and specifically of *A.muciniphila* and at the phylum level, the ratio of Firmicutes to Bacteroidetes was reduced. Evidence was also obtained that bile acid concentration was higher in the ileum of bean-fed mice but that the levels of FXR transcripts and its downstream targets were unaffected. Whether alterations in bile acid metabolism exert other functional consequences relative to fat accumulation and/or overall metabolic health requires further investigation. 

## Figures and Tables

**Table 1:** Composition of Experimental Diets.

Ingredient	Low Fat Control Diet ^1^ (g/100g)	High Fat Control Diet ^1^ (g/100g)	Cooked, Whole White Kidney Bean ^1^ (g/100g)
Solka-Floc	4.7	6.5	0.0
White kidney bean	0.0	0.0	40.0
Corn Starch	29.9	0.0	0.0
Casein (≥85% protein)	19.0	25.8	17.1
Cerelose (Dextrose)	3.3	16.2	0.0
Sucrose	33.2	8.9	0.3
Vitamin mix ^2^	1.0	1.3	1.3
DL-Methionine	0.3	0.4	0.4
L-Tryptophan (Sigma T0254-25G)	0.00	0.00	0.01
Choline bitartrate (41% choline)	0.2	0.3	0.3
Mineral mix ^3^	4.3	5.8	5.8
Soybean oil	2.4	3.2	3.2
Palm Oil	1.9	31.7	31.7
TOTAL (g)	100.0	100.0	100.0

^1^ Experimental diets modified from the original diet formulations. The experimental diets used ^2^ Dyets #310025 AIN-93G vitamin mix and ^3^ Dyets #210025 AIN-93G mineral mix.

**Table 2:** qPCR primers.

**Gene Expression Primers**	**Sequence**	**References**
18S FWD	5′- ATTGGAGCTGGAATTACCGC -3′	[19]
18S REV	5′- CGGCTACCACATCCAAGGAA -3′	[19]
FXR FWD	5′- TGGGCTCCGAATCCTCTTAGA -3′	[19]
FXR REV	5′- TGGTCCTCAAATAAGATCCTTGG -3′	[19]
SHP FWD	5′- TCTGCAGGTCGTCCGACTATTC -3′	[19]
SHP REV	5′- AGGCAGTGGCTGTGAGATGC -3′	[19]
FGF15 FWD	5′- GCCATCAAGGACGTCAGCA -3′	[19]
FGF15 REV	5′- CTTCCTCCGAGTAGCGAATCAG -3′	[19]
**Bacterial Primers**	**Sequence**	**References**
16S (926) FWD	5′- AAA CTC AAA KGA ATT GAC GG -3′	[21,22]
16S (1062) REV	5′- CTC ACR RCA CGA GCT GAC -3′	[21,22]
*Akkermansia Muciniphila* FWD	5′- CAG CAC GTG AAG GTG GGG AC -3′	[23]
*Akkermansia Muciniphila* REV	5′- CCT TGC GGT TGG CTT CAG AT -3′	[23]
Bacteroidetes FWD	5′- AAA CTC AAA KGA ATT GAC GG -3′	[24]
Bacteroidetes REV	5′- GGT AAG GTT CCT CGC GCT AT -3′	[24]
Firmicutes (928) FWD	5′- TGA AAC TYA AGG AAT TGA CG -3′	[24]
Firmicutes (1040) REV	5′- ACC ATG CAC CAC CTG TC -3′	[24]

Forward: FWD; Reverse: REV; farnesoid X receptor: FXR; small heterodimer partner: SHP; fibroblast growth factor 15: FGF15.

**Table 3:** Effect of white kidney bean on food efficiency ratio and fecal energy (Experiment 2).

Diet ^1^	Total Diet Eaten (g)	Total Weight Gained (g)	Feed Efficiency Ratio	Total Feces Excreted (mg/day)	Fecal Energy Concentration (kcal/g)	Total Fecal Energy/day (kcal)
Control	154.5 ± 6.6	3.5 ± 1.8	0.023 ± 0.012	256.9 ± 18.4	3.77 ± 0.11	0.97 ± 0.09
Bean	167.3 ± 16.9	2.8 ± 1.7	0.016 ± 0.009	273.8 ± 33.7	3.77 ± 0.05	1.03 ± 0.13
*p*-value	0.0650	0.4467	0.2412	0.2350	0.9987	0.2601

^1^ Values are means ± SD; Control *n* = 8, Bean *n* = 8.

**Table 4:** Effect of white kidney bean on body weight and adiposity (Experiment 2).

Diet ^1^	Final Body Weight (g)	Body Mass Index ^2^ (g/mm)	Subcutaneous Fat Mass ^3^ (mg/mm)	Sum Visceral Fat Mass ^4^ (mg/mm)	Total Fat Mass ^5^ (mg/mm)
Control	38.2 ± 3.3	2.2 ± 0.2	64.1 ± 9.1	189.1 ± 30.3	253.2 ± 38.3
Bean	38.4 ± 3.4	2.2 ± 0.2	47.6 ± 12.2	172.5 ± 33.3	220.1 ± 44.3
*p*-value	0.9182	0.9486	0.0085	0.3138	0.1323

^1^ Values are means ± SD. ^2^ Units are g or mg mass divided by length of tibia in mm; ^3^ Inguinal fat; ^4^ Sum total of mesenteric fat, retroperitoneal fat and epididymal fat depots. ^5^ Sum total of inguinal subcutaneous and sum visceral fat depots; Control *n* = 8, Bean *n* = 8.

**Table 5:** Effect of feeding of white kidney bean on intestinal morphometry (Experiment 2).

Measurement ^1.^	Tissue	HF Control	Bean	*p*-Value
Crypt height (µm^2)^	Ileum	252.1 ± 41.1	274.5 ± 29.5	0.2916
	Ascending colon	92.2 ± 9.3	82.2 ± 11.7	0.1141
	Transverse colon	182.2 ± 23.7	214.1 ± 13.1	0.0136
	Descending colon	135.9 ± 9.6	153.9 ± 16.6	0.0324
Alcian blue area (µm^2^)	Ileum	1170.5 ± 557.5	1105.3 ± 280.1	0.8008
	Ascending colon	1335.5 ± 248.7	1215.1 ± 312.6	0.4549
	Transverse colon	4344.1± 1274.9	5711.3 ± 1.518.2	0.1049
	Descending colon	1731.4 ± 360.6	1508.1 ± 263.1	0.2358
Ki-67 (%) ^2^	Ileum	26.3 ± 5.8	26.4 ± 4.8	0.9673
	Ascending colon	19.4 ± 3.4	21.7 ± 3.2	0.2451
	Transverse colon	19.1 ± 5.9	22.9 ± 3.4	0.1908
	Descending colon	17.6 ± 2.0	17.5 ± 1.3	0.8658

^1^ Values are means ± SD; ^2^ The percentage of Ki-67 positive cells were measured along the entire length of each crypt; High fat (HF) control *n* = 7, Bean *n* = 6.

## Supplementary Materials

The following are available online at https://www.mdpi.com/2072-6643/11/11/2780/s1, Figure S1: Metabolic cage design.
