# Supplementary material for: White Kidney Bean (Phaseolus Vulgaris L.) Consumption Reduces Fat Accumulation in a Polygenic Mouse Model of Obesity
Source: Nutrients. 2019 Nov 15;11(11):2780. doi: 10.3390/nu11112780 (PMC6893514; doi:10.3390/nu11112780)
Supplement: Supplementary file 1 [file nutrients-11-02780-s001.zip › Supplementary Figure S1 - Metabolic Cage Design.docx]

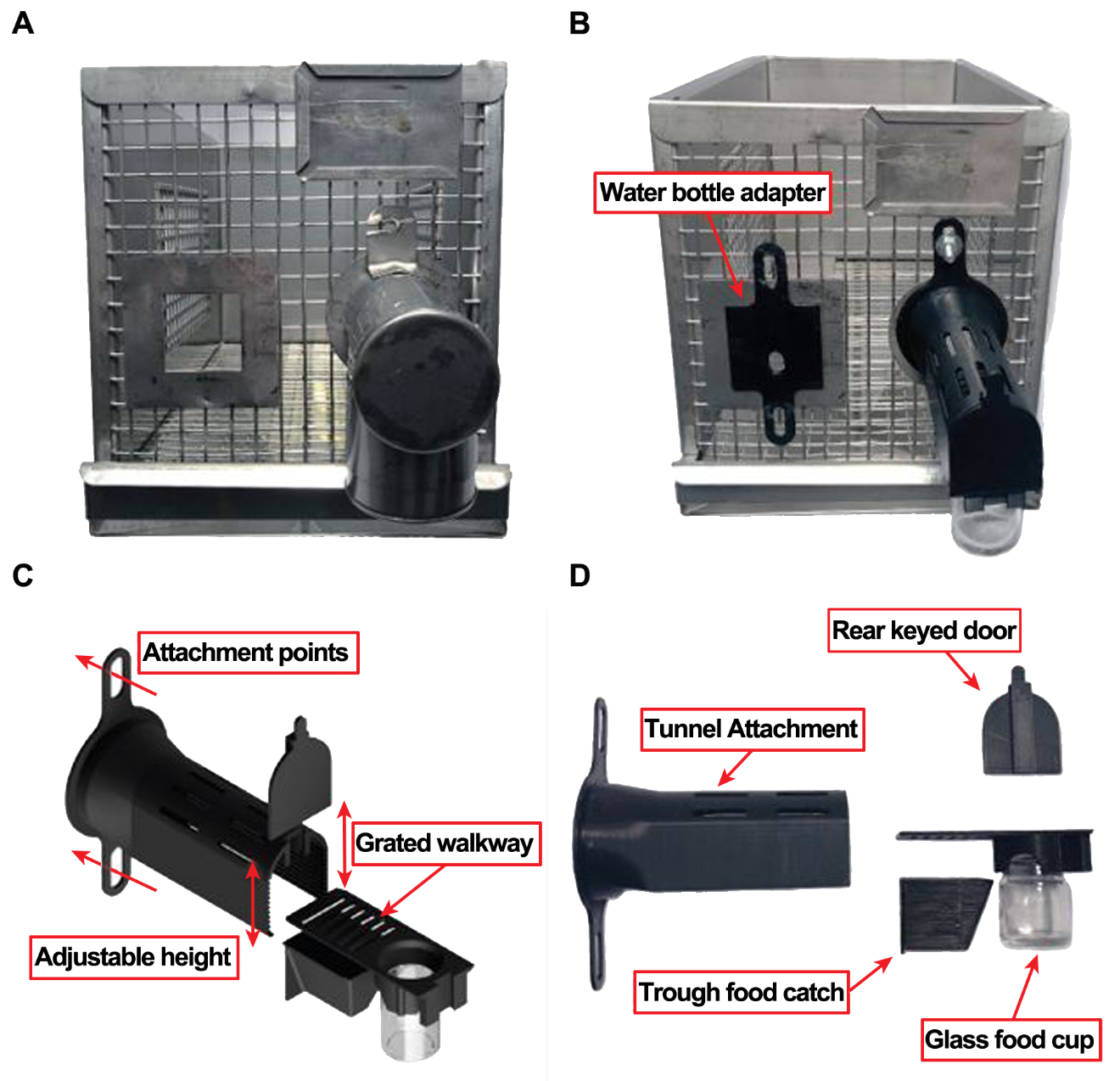


**Supplementary Figure S1.** Modified tunnel feeders created in house. (**A**) As-purchased metabolic rat cage with stainless steel feeding tunnel and collection funnel. (**B**) Modified metabolic cage for use with mice. Custom-made feeding tunnel and water bottle adapter are attached to the cage via bolts and nuts. A small polycarbonate plastic piece was placed inside the cage to be used as a resting platform for the mice. (**C**) 3D-render of the new tunnel feeders. The adjustable height allows for animal-specific settings. The proper setting keeps feeding quarters tight, preventing the mice from easily turning around to bring food to burrow. The grated walkway causes the animals to drop any food they attempt to nest with. (**D**) The tunnel feeders were 3D printed using black FDA approved food-safe PLA. There was no evidence of the mice chewing on the plastic. The glass food cups snap-fit into the grated walkway and the trough collects any dropped food and feces. The rear door slides in preventing animal escape.
